# Air Pollution Exposure in Walking School Bus Routes: A New Zealand Case Study

**DOI:** 10.3390/ijerph15122802

**Published:** 2018-12-10

**Authors:** Kim N. Dirks, Jennifer A. Salmond, Nicholas Talbot

**Affiliations:** 1 School of Population Health, Faculty of Medical and Health Sciences, University of Auckland, Auckland 1142, New Zealand; 2 School of Environment, Faculty of Science, University of Auckland, Auckland 1142, New Zealand; j.salmond@auckland.ac.nz; 3 Research and Investigations, Auckland Council, Auckland 1010, New Zealand; nick.talbot@aucklandcouncil.govt.nz

**Keywords:** walking school bus, air pollution exposure, safety, children, traffic

## Abstract

Walking School Buses (WSBs), organized groups for children to walk to school under the supervision of adults, help reduce traffic congestion and contribute towards exercise. Routes are based largely on need, traffic safety and travel time, with exposure to air pollution not generally considered. This paper explores whether reductions in exposure can be achieved based on the side of the road travelled using data collected in Auckland, New Zealand. Exposure to air pollution was measured for a 25-min commute consisting of a 10-min segment along a quiet cul-de-sac and a 15-min segment along a main arterial road with traffic congestion heavier in one direction. Two participants were each equipped with a portable P-Trak ultrafine particle monitor and a portable Langan carbon monoxide monitor, and walked the route on opposite sides of the road simultaneously, for both morning and afternoon, logging 10-s data. The results suggest that pedestrians travelling on the footpath next to the less congested side of the road in the morning avoid many short-term peaks in concentration and experience significantly lower mean exposures than those travelling on the footpath next to the more congested side. Significant reductions in air pollution exposure could be made for children by taking into account the side of the road in WSB route design.

## 1. Introduction

Regular engagement in physical activity is vital for good health and well-being. The New Zealand Ministry of Health recommends that children aged 5 to 16 years engage in at least one hour of moderate or vigorous aerobic exercise each day and that children stay active as much as possible, including when travelling from place to place [1]. Walking to school is one way to help children reach their daily recommended level of physical activity and generally help them to stay active. Other benefits of walking to school include the enjoyment of it, the opportunity for increased social cohesion with classmates, the opportunity to learn about road traffic safety, the encouragement of walking as a normal activity [2], as well as reducing traffic congestion around schools [3]. While many children are now driven to school, there is evidence that many children from cities, including those in the UK and in New Zealand, would prefer to walk to school rather than be driven if given the opportunity to choose [2,4].

Despite all of the benefits, the uptake of walking to school remains relatively low across much of the developed world. For example, in Auckland, New Zealand, 40% of primary school aged children walk to school [5], despite most children living in very close proximity to their local school. It has also been noted that children more often walk home from school in the afternoon than walk to school in the morning. This is believed to be due to the morning commute being more often linked to the parents travelling to work [2].

A significant literature now exists around the barriers associated with walking to school amongst primary school-aged children. This includes studies into the risk of traffic-related accidents, as well as concerns around ‘stranger danger’ [6]. The need for solutions to alleviate these fears has led to the development of organized walking groups, coined walking school buses (WSBs), or Walk to School programmes, whereby children walk in groups to school, accompanied by adults, along a pre-specified route chosen based largely on catchment need, traffic safety considerations, parental participation and motivation to ‘drive’ the school buses. Such groups have become popular around the world, including in the UK where they were first proposed [7], as well as across New Zealand [6]. As of 2006, 83 schools across New Zealand had at least one WSB route registered, with participating schools tending to be concentrated in the wealthier neighborhoods [3].

One factor generally not taken into account in walking school bus route planning is the air pollution exposure experienced by children during their commute. There is mounting evidence in the literature of the association between exposure to traffic-related pollution and adverse health outcomes, including cancer [8]. Traffic air pollution has also been linked to a range of respiratory-related conditions, including the exacerbation of asthma amongst both children and adults [9]. While all people are at risk when exposed to traffic pollutants, children have been found to be more susceptible to adverse effects than adults [10].

Given that traffic-related air pollution exposure has been linked to both acute health effects triggered by short periods of elevated exposures, and chronic health effects resulting from cumulative effects over extended periods of time [8], an effort should be made to avoid both peaks in exposure as well as minimizing the cumulative exposures experienced during high exposure activities such as commuting. In the case of walking to school, children have the potential to be exposed to elevated levels of air pollution over a long period of time (five days a week for many days of the year over many years) at a time in their lives when they are most vulnerable. The close proximity to roads and the lack of a physical barrier between exhaust pipes and respiratory systems also make pedestrians vulnerable to peaks in air pollution concentrations [11].

Concentrations of traffic-related air pollutants along road corridors are influenced by the traffic flow rate, state of congestion, as well as the local meteorology, including the wind speed and direction. In relation to the commute to school, significantly higher exposures can be expected for children walking to school in the morning compared with during their journey home because of the timing in relation to ‘rush hour’ traffic and also because of the more effective dispersion conditions that tend to exist in the middle of the afternoon compared with the early morning due to higher wind speeds.

The air pollution doses experienced by commuters travelling along a given route will depend not only on the pollutant concentrations in the road corridor but also the time spent commuting. The separation of the commuter relative to the main line of traffic has been found to have a significant influence on exposure with the larger the separation from the traffic, the lower the exposure [12]. In fact, a recent study carried out on a busy street in London showed that pedestrians travelling at the building edge of the footpath within a street canyon experienced significantly lower exposures than those walking on the footpath close to the road edge [13]. While for some journeys there are options available for avoiding congested roads altogether, other journeys involve roads with congestion that are impossible to avoid, for example, in the case of a school located along a main arterial road with that road being the only access to the school. In this case, especially if the route is dominated by traffic in one direction (‘rush hour’ traffic), the side of the road on which children walk could have a significant impact on their air pollution dose because of the difference in the separation of the walker from the lane of traffic with the greatest amount of congestion.

The aim of this paper is to determine, through field work, the extent to which the air pollution doses (in this case, ultrafine particle (UFP) counts, and carbon monoxide (CO) concentrations) of walk-to-school commuters can be reduced by the careful selection of the best side of the road to travel to avoid walking on the footpath closest to the more congested side of the road. This has been carried out by measuring the air pollution exposure for two hypothetical walk-to-school commuters travelling on either side of a road simultaneously on walks both to school and on the journey home. Any strategy for reducing air pollution exposure while ensuring children continue to walk (thereby receiving the exercise and other advantages of walking) would be beneficial, both for children as well as adults partaking in the walk to school. Significant differences in exposure patterns could have implications for the planning of WSB routes to ensure the best possible environment for children partaking in walking.

## 2. Materials and Methods

### 2.1. Route

**Figure 1:**
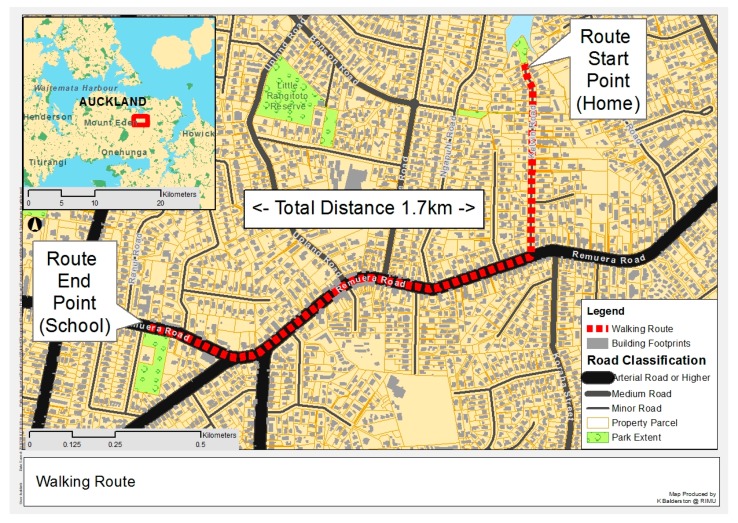
Walking route. Note the location of the start of the walking route (home) as well as the end (school) for the morning commute and the reverse for the journey home.

A route of 1.7 km in length was chosen for a school located in the inner city suburb of Remuera in Auckland. The route (shown in Figure 1) consists of a short walk (10 min) along a quiet cul-de-sac segment (Kelvin Road), followed by a slightly longer segment of 15 min along a main arterial road (Remuera Road), consisting of many school and city buses as well as cars, until just before Ranui Rd where the school is located. There are footpaths on both sides of the street and several intersections and signalized pedestrian crossings along the route where school children can cross the main road safely. There are many schools in the area and many children catch school buses at stops along Remuera Road to get to schools located in other nearby suburbs.

### 2.2. Traffic

For Remuera Road, the main arterial road, traffic data are available from Auckland Council since 2009 at two locations: one near Waiatarua Road just off the end of the start of the Remeura Road segment of the WSB route, and one near Ranui Road, near the main entrance to the school. From these data, the traffic flow rates and the proportion of traffic flow for each direction were identified for both the morning and evening rush hour periods. No Council traffic data are available for Kelvin Road. However, traffic flows are very light at the time of the morning and afternoon commutes, with, typically, one or two cars passing during the 10 min walking time to or from the main arterial road segment.

### 2.3. Data Collection and Instrumentation

Ultrafine particle (UFP) counts and carbon monoxide (CO) were chosen as the pollutants of interest. Low-cost and reliable portable air pollution monitoring exists for these two pollutants and each has been used in previous studies [11,12,13] as a marker for diesel (UFP) and petrol (CO) emissions. For the field trials, two participants were each equipped with a P-Trak ultrafine particle monitor (TSI Instrument) and a Langan portable carbon monoxide monitor (Langans Products, Inc) to measure personal air pollution exposures during their commute to and from school. The participants walked the route on opposite sides of the road for most of the journey, and, as much as possible, remained aligned with one another so they arrived at the destination at the same time. Air pollution data were logged at ten-second intervals over the period of the commute for a total of nine commutes in the morning and three in the afternoon. Rainy days were excluded as the equipment is not able to be operated under wet conditions. Sampling began at the beginning of the route at 7:40 am each day and finished around 8:05 am at the school and then from 3:10 pm until about 3:35 pm for the afternoon commute home. For the ‘parent’ return journey (home in the morning and to school in the afternoon), the monitors were transported by a single person to allow for a comparison between monitors (a co-location). Data collection took place between 10 and 23 October 2015. The exact time of departure, arrival and crossing of roads (kept as consistent as possible) were noted for partitioning data for analysis.

### 2.4. Meteorological Data

Meteorological data were obtained from the National Institute of Water and Atmospheric Research (NIWA) climate database. The Penrose site was selected as the most suitable based on the method recommended by the study of Elangasinghe et al. (2016) [14] for meteorological station site selection for air pollution studies. The mean wind speed, wind direction, temperature and humidity were retrieved for the three 10-min periods closest to which the commutes took place (7:50–8:10 am for the morning commutes and 3:10–3:30 pm for the afternoon commutes).

### 2.5. Data Analysis

Time series of exposures were created for all of the commutes for both carbon monoxide and ultrafine particle concentrations for each side of the road. Average commuter exposures were calculated for each journey for each day for the complete route, as well as separately for the main arterial road segment. Route averages were then compared between the morning and afternoon commutes, and between sides of the road separately for the morning and afternoon commutes and for each of the two pollutants measured, using unpaired t-tests. Statistical significance was set at a value of 0.05.

For the analysis of peak concentrations, peak events were set arbitrarily as those above 100,000 counts/cm^3^ for UFP and 2 ppm for CO. These were noted and classified based on time of day and side of the road on which the peak was recorded. These peaks were then ranked and plotted in order of decreasing concentration for the purpose of highlighting differences in the occurrence of peak concentrations that were experienced during journeys.

## 3. Results

### 3.1. Traffic Data

The most recent traffic data available for the two sites along the main arterial segment of the walking school bus route (Ranui Road and Waiatarua Road) sorted by direction of flow and time of day (morning and evening peak), collected in 2012, are shown in Table 1. Note that no data are available specifically for the end-of-school period. While varying from site to site, traffic flows for the two directions combined are about 2000 veh/h along the main arterial road of the walking school bus route at peak times of 8:00–9:00 am and 5:00–6:00 pm. During the morning, outside of the school, there is approximately twice the traffic flow heading towards Auckland City Centre westward on the south side of the road compared with traffic heading away from Central Auckland, eastward on the north side. During the evening peak, these is an almost equal traffic flow rate in either direction. For the site located closer to the home end of the main arterial road, 60% of the traffic is city bound in the morning and 60% is outbound in the evening.

Data collected at the Waiatarua Road intersection in 2015, the most recent data for which traffic composition information are available, indicate that approximately 95% of the traffic flow along the main arterial road (Remuera Road) consist of cars. Approximately 5% are heavy commercial vehicles, all of which can be expected to be diesel vehicles, with observations suggesting that these consist mainly of city and school buses.

### 3.2. Meteorological Data

**Figure 2:**
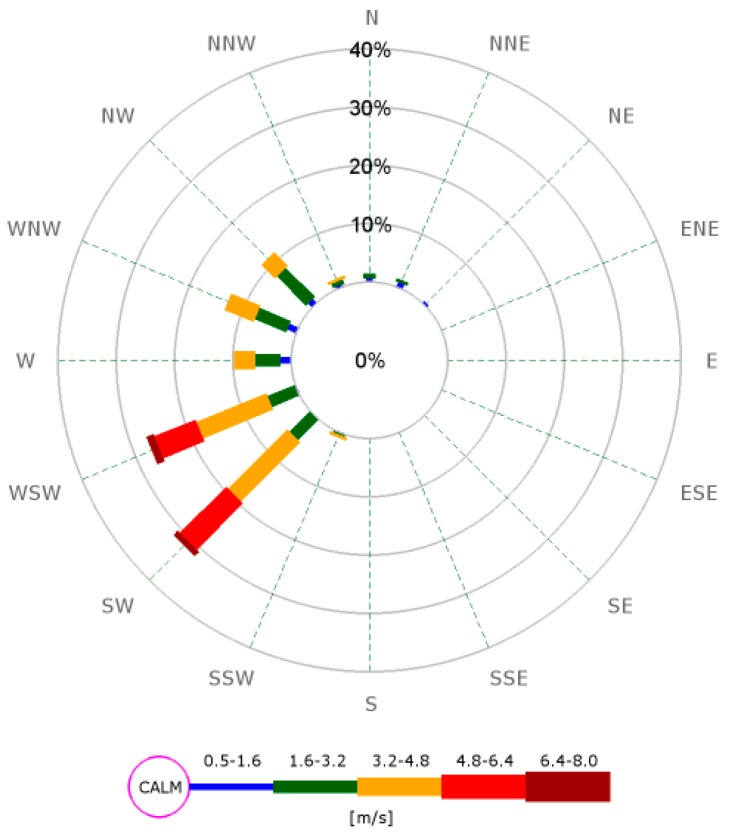
A wind rose created from data taken from the Penrose air quality and meteorological monitoring station run by Auckland Council covering the period 12–24 October 2015.

Table 2 shows the meteorological data for the 12 periods of data collection (nine morning commutes and three afternoon commutes). The data are based on the three 10-min averages most aligned with the commuting periods: 7:50–8:10 am and 3:10–3:30 pm representing the morning and afternoon commutes, respectively. The data are from the Penrose site located just over 3 km away from the location at which the field trials were carried out. Note that the wind direction and temperature varied little throughout the period of observation. The commuters on the footpath closest to the more congested side of the road were thus windward of the road most of the time in the morning and leeward on the way home. The wind speed was more variable, ranging from 2.1 m/s to 5.1 m/s based on 10-min averages. The wind rose shown in Figure 2 emphasizes the west and southwesterly flow of air during the periods of observation.

### 3.3. Air Pollution Exposure Data

**Figure 3:**
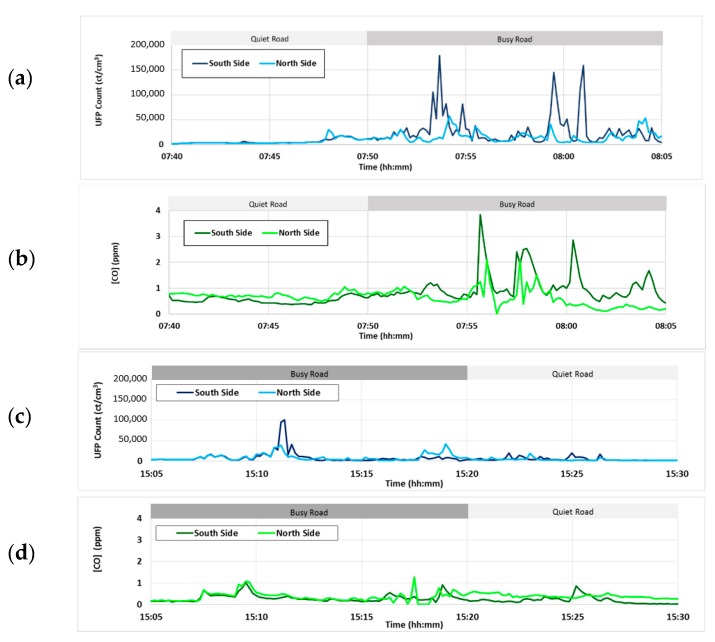
Example time series of exposure during the morning Walking School Bus (WSB) for (**a**) UFP (ultrafine particle) and (**b**) CO and the afternoon (**c**) UFP and (**d**) CO. The participant walking on the north side of the street (high traffic side during the morning rush hour and low in the afternoon) is shown in the darker color.

The descriptive statistics for each time of day and side of the road travelled are presented in Table 3. Complete route average UFP counts range from 6900 counts/cm^3^ to 39,900 counts/cm^3^ while complete route average carbon monoxide concentrations range from 0.24 ppm to 1.24 ppm. The results show significant day-to-day variability in mean concentrations for the two pollutants monitored, related to the changing prevailing meteorological conditions at the time of sampling. Figure 3 shows examples of time series of pollution concentrations for both a morning and an afternoon commute, for both ultrafine particle concentrations and carbon monoxide. Note that concentrations in the morning remain relatively low for the first 10 min of the commute along the quiet cul-de-sac segment (Kelvin Road) and then increase significantly around 7:50 am when the pedestrians start their journey along the main arterial road segment (Remuera Road). In the afternoon, concentrations tend to remain significantly lower than in the morning, and very low when the commuters reach the quiet cul-de-sac segment around 3:20 pm. Peaks in concentration are observed for both ultrafine particle and carbon monoxide concentrations in the morning, especially for the walker travelling on the footpath near the more congested side of the road with traffic heading into the city, and when the pedestrians crossed over the major intersection located halfway along the main arterial road segment (not shown). 

The results of the study show that both the average ultrafine particle concentrations and carbon monoxide concentrations for the morning commute to school were significantly higher than for the commute home for both UFP (*t* = 4.03, *p* = 0.002 and *t* = 5.855, *p* <0.001) for travel on the North and South side, respectively, and for CO (*t* = 2.80, *p* = 0.019 and *t* = 2.33, *p* = 0.042) for travel on the North and South side, respectively, based on data for the complete route.

**Figure 4:**
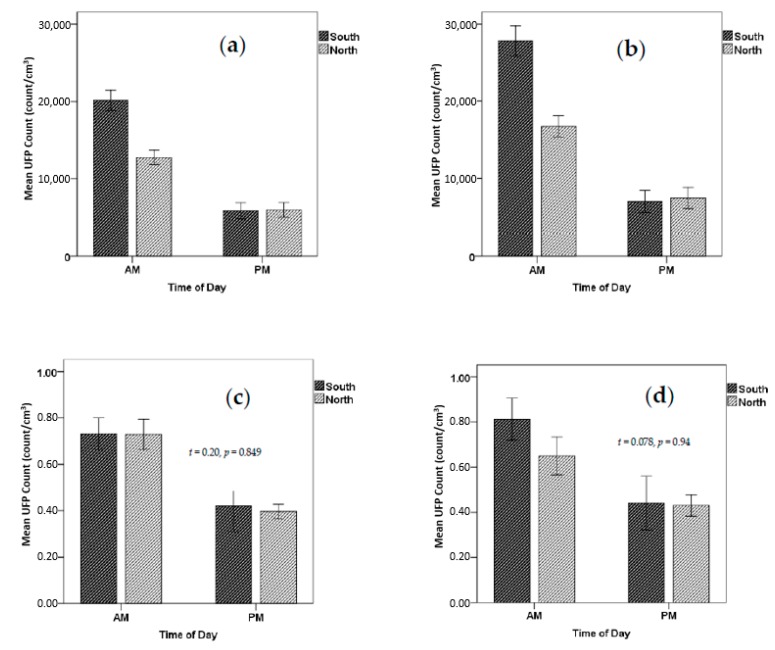
Mean concentrations for each time of day and route (**a**) When travelling the complete route: UFP, (**b**) For the main arterial road segment only: UFP, (**c**) When travelling the complete route CO, (**d**) For the main arterial road segment only: CO (error bars are one standard error of the mean (SEM)).

Figure 4 compares the commute average exposures for the two sides of the road (South and North) for both ultrafine particle counts and carbon monoxide; Figure 4a,c are for the complete route while Figure 4b,d are for the main arterial road segment. Note that significantly higher mean UFP concentrations were observed when travelling along the South side of the road (adjacent to the dominant traffic), both for the complete route (a factor of 1.57, *t* = 4.57, *p* <0.001) and for the main arterial road segment only (a factor of 1.65, *t* = 4.62, *p* <0.001). This was not the case for the commute home when average concentrations were found to be not significantly different for either the complete route or for the main arterial road only segment. In contrast, no significant differences were found between average carbon monoxide exposures, either for the morning or the afternoon commutes, for either the complete route or for the main arterial road segment only.

### 3.4. Exposure to Peaks in Concentration

**Figure 5:**
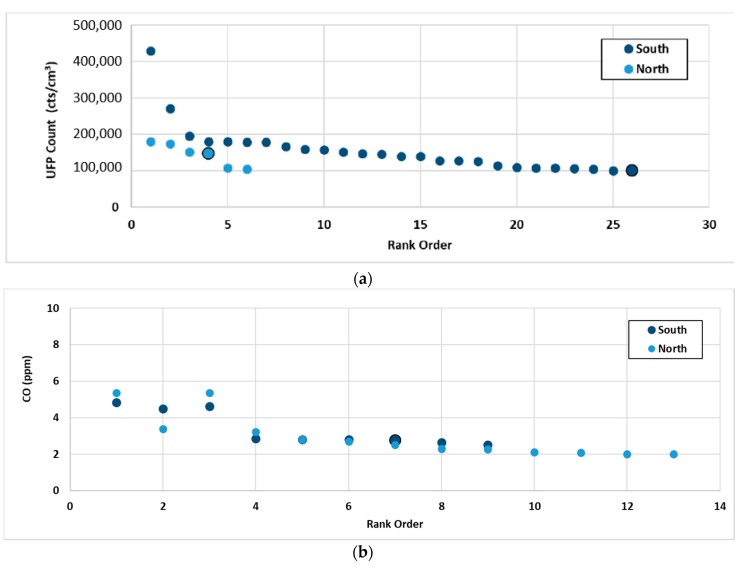
Rank order of largest peaks in concentration for the morning commute for (**a**) UFP and (**b**) CO. Large markers indicate peaks that have occurred in the afternoon—all of the rest occurred in the morning.

Peak concentrations are of interest as they have the potential to trigger respiratory episodes amongst those who are vulnerable. Figure 5 shows the 10-s average peak values of all episodes in which the peak concentrations of ultrafine particles exceeded 100,000 counts/cm^3^, stratified based on the side of the road travelled by the commuter and then ranked from highest to lowest. Of the 32 peaks observed, all occurred on the main arterial road. Of these, 26 occurred when the walker was travelling on the footpath located closest to the more congested side (South in the morning and North in the afternoon), and 30 of the 32 occurred during the morning commute. Note that data were sampled at a 3:1 morning-to-afternoon ratio, suggesting that the likelihood of a peak event is about three times that in the morning compared with the afternoon. For CO, the side of the road had little impact on the likelihood of exposure to a peak event or the magnitude. It is not clear why this is the case and further exploration is required. While not shown here, the peaks in CO did not generally coincide with the peaks in UFPs, indicating that different vehicles and engine types were associated with the peaks (diesel for the UFP and petrol for the CO).

## 4. Discussion

**Figure 6:**
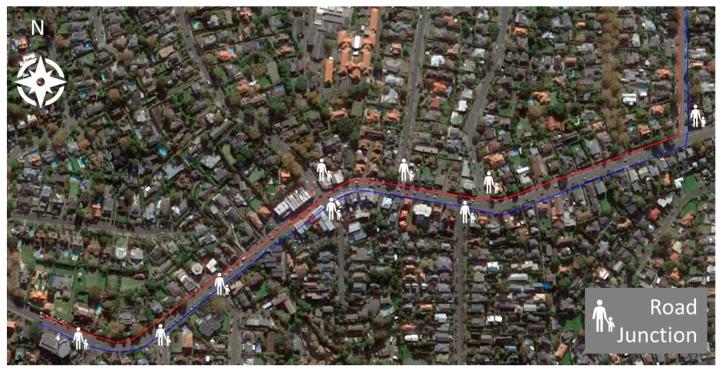
WSB (Walking School Buses) route. The red line shows the north side of the footpath and the blue line shows the south side of the footpath. The places where pedestrians have to cross roads are shown.

A London exposure study [13] found that the path chosen along a specific footpath had a significant impact on the air pollution exposures of pedestrians. This is supported by the study undertaken by Grange et al. (2014) [12] and others suggesting that even a modest increase in separation between traffic and active mode commuters can lead to a significant reduction in air pollution exposure. In the present study, pedestrians who travel on different sides of a road have been compared, with implications for the design of walking school bus routes for children on the way to and from school with respect to both peak and mean concentrations. A study in Spain identified diesel buses as the key source of UFP within their study. The same report also shows that the highest spikes in concentration were recorded at road junctions where vehicles were idling [15]. Figure 6 shows the number of junctions where the WSB would have to wait to cross roads. The bus on the south side of the road must cross six roads compared to three for the north side. The number of junctions might also have increased the likelihood of receiving spikes in UFP and CO.

The journey average concentrations (Table 3) are consistent with other Auckland-based studies investigating pedestrian exposures [11,12]. For example, average UFP counts for the study carried out by Dirks et al. (2012) [11] were 15,370 counts/cm^3^ and 15,267 counts/cm^3^ for pedestrians on their side of the street compared to an overall mean of 12,938 counts/cm^3^ in the present study. Significant increases in exposure when travelling on the more congested (south side) of the road were found for ultrafine particle concentrations during the morning commute when 60% of the traffic and most of the buses were city-bound, but not for carbon monoxide. This could be a reflection of the dominance of buses travelling city-bound in the morning; the vehicles contributing to ultrafine particle counts, but not to petrol-engine vehicles that are responsible for most of the carbon monoxide. No significant differences were found between sides of the road in terms of exposure for either ultrafine particle counts or carbon monoxide concentrations for the afternoon period. Peaks in ultrafine particle exposure were observed mainly during the morning commutes and also for commuters walking on the more congested (South) side of the road at that time of day.

Previous research carried out in Auckland has identified transport as the major contributing factor to PM_2.5_ measurements [16] whilst buses were found to contribute to plumes of UFP pollution along footpaths [17]. Moreover, the distance from the roadside and the height of the pedestrian will also impact upon personal exposure [18], with plume density (and therefore UFP number concentrations) higher closer to the road and lower to the ground.

This study suggests that travel along congested roads should be avoided in favor of travel along quieter streets, and that attention should be paid to the side of the road on which children walk. This will ensure that long-term exposure will be minimized as well as the likelihood of exposure to peaks in concentration.

Exposures were observed to be considerably lower in the afternoon compared with the morning. There are several mechanisms that might explain this. A noticeable increase in wind speed during the afternoon period (Table 2) would help remove locally-emitted pollutants more rapidly than during the morning walk. Increased vertical mixing due to increased boundary layer height would also increase dilution of pollutants during the afternoon period. The number of buses in the afternoon is also reduced, helping with this reduction. The differences in exposures for the two sides of the road is also reduced in the afternoon. This can be explained, at least in part, by the evening traffic which is less dominated by flow in one direction at that time of the day compared with the morning period. The number of buses and the flow of PM traffic might also play a part in reducing concentrations. The fact that more children walk home from school [2] rather than to school in the morning is helpful in terms of maximizing the benefits of exercise, social interaction and learning about traffic safety, while minimizing the adverse effects of air pollution exposure.

There are several limitations associated with this study. Firstly, only one route was investigated and over only a short two-week period during which time the range of meteorological conditions was relatively narrow. Every route is unique in terms of its traffic flows, traffic light phasing and local meteorology, so exposures can be expected to vary considerably from day to day and season to season. None-the-less, significant patterns emerged over the period of sampling leading to statistically significant differences in exposure due to the side of road travelled for UFP.

While the traffic data were able to reveal the proportion of heavy-duty vehicles travelling along the road, the proportion of heavy-duty traffic was not sorted by direction. As such, it was not possible to estimate the proportion of heavy-duty vehicles travelling on the more congested side of the road compared with the less congested side. Also, the traffic flows were only available as 24-h counts or by hour at peak times, in this case 5:00 pm–6:00 pm rather than 3:00 pm–4:00 pm when children travel home from school. None-the-less, observations made during the study suggest that the heavy-duty traffic consisted mostly of buses and that the majority were heading in the direction of the city during the morning commute to school, impacting on ultrafine particle concentrations and not so much on carbon monoxide.

## 5. Conclusions

The focus of this study has been on WSB routes as they are planned in a way that is more prescribed compared with many other walking journeys. However, it is important to note that the results of this study apply to any pedestrian route, whether walking to school or not, or whether walking in an organized group or not.

There are many factors that should be considered when planning a walking route to school for children, including pedestrian safety with respect to road accidents. However, air pollution should also be considered. The results from this study identified time of day, traffic volume with specific focus on bus concentrations, as well as the number of junctions a pedestrian might have to cross to be the main factors in deciding personal exposure to potentially hazardous air pollutants. The implications of these key findings suggest that congested roads should be avoided if possible, in favor of quieter roads to minimize personal exposure. If travel along congested roads is unavoidable, then consideration should be made of travel along the footpath on the side of the road furthest from the lane of traffic with the most congestion. Pedestrians travelling on the side opposite the more congested side avoid many short-term peaks in concentration and experience significantly lower mean exposures than those travelling on the footpath closest to the more congested traffic.

However, route planning decisions need to be weighed up against the extra time spent waiting at intersections and pedestrian crossings as pollution levels tend to be higher there than along the segment between intersections [19]. The increased risk of accidents associated with crossing the road and the availability of suitable places to cross also need to be taken into account. 

There are numerous benefits to encouraging children to walk to school. Parents should be encouraged to contribute to WSBs and other initiatives aimed at facilitating the opportunity for children to walk to school. Route choices should be based on consideration of air pollution exposure as well as catchment needs, traffic safety and travel time efficiency to maximize the health benefits and minimize the risk.

## Figures and Tables

**Table 1:** Traffic volume by flow and direction along the main arterial road (Remuera Road) at the Waiatarua and Ranui Road crossings for the morning commuting peak hour (am 8:00–9:00) and afternoon commuting peak hour (pm 5:00–6:00) periods.

Location	Time	Total Flow for Road Segment (veh/h)	Direction	Traffic Flow (veh/h)	Percentage Flow by Direction
Waiatarua Road	am	1920	West	1111	58%
			East	809	42%
	pm	2171	West	822	38%
			East	1349	62%
Ranui Road	am	1884	West	1238	66%
			East	646	34%
	pm	1951	West	954	49%
			East	997	51%

**Table 2:** Key meteorological data including means and standard deviations obtained from the Penrose air quality monitoring station situated just over 3 km to the south of the experimental site. The values are based on the three 10-min average observations made closest to the commute time (7:50–8:10 am and 3:10–3:30 pm).

Date	am/pm	Wind Speed (mean ± stdev) (m/s)	Wind Direction (mean ± stdev) (degrees)	Temperature (mean ± stdev) (°C)	Humidity (mean ± stdev) (%)
12/10/2015	am	4.0 ± 0.6	249 ± 1	13.4 ± 0.5	86 ± 3
13/10/2015	am	2.1 ± 0.2	318 ± 6	14.1 ± 0.2	77 ± 1
15/10/2015	am	4.0 ± 0.6	227 ± 8	13.1 ±0.1	63 ± 1
16/10/2015	am	3.9 ± 0.3	230 ± 11	13.0 ± 0.1	68 ± 0
19/10/2015	am	5.1 ± 0.4	250 ± 3	13.3 ±0.1	64 ± 1
20/10/2015	am	4.3 ±0.4	240 ± 2	12.5 ± 0.3	64 ± 4
21/10/2015	am	2.9 ± 0.8	258 ± 28	13.4 ± 0.3	75 ± 3
22/10/2015	am	2.9 ± 0.5	296 ± 7	15.5 ± 0.2	77 ± 1
23/10/2015	am	2.6 ± 0.2	287 ± 9	16.2 ± 0.1	77 ± 1
12/10/2015	pm	5.5 ± 0.6	235 ± 2	16.5 ±0.2	57 ± 1
13/10/2015	pm	3.6 ± 0.4	276 ± 10	16.5 ± 0.1	62 ± 1
20/10/2015	pm	5.3 ± 0.5	243 ± 3	14.7 ± 0.3	54 ± 2

**Table 3:** Descriptive statistics for air pollution exposures on walking journeys. ‘All’ represents the complete route while ‘Main Arterial’ is restricted to observations made when travelling on the main arterial road segment.

Data	am/pm	Side of Road	*N*	Min UFP (counts/cm^3^)	Max UFP (counts/cm^3^)	Mean UFP (counts/cm^3^)	STDEV UFP (counts/cm^3^)	Min CO (ppm)	Max CO (ppm)	Mean CO (ppm)	STDEV CO (ppm)
All	am	South	9	14,600	28,000	20,100	1300	0.43	1.00	0.73	0.07
		North	9	9800	18,800	12,800	900	0.47	1.07	0.73	0.07
	pm	South	3	4100	7700	5800	1000	0.24	0.62	0.42	0.11
		North	3	4100	6900	5900	900	0.36	0.46	0.39	0.32
Main Arterial	am	South	9	21,100	39,900	27,800	1900	0.40	1.12	0.81	0.09
		North	9	12,300	26,600	16,800	1400	0.35	1.24	0.65	0.08
	pm	South	3	4800	9600	6900	1400	0.25	0.66	0.44	0.12
		North	3	4700	9200	7400	1400	0.36	0.52	0.43	0.05

UFP: ultrafine particle, CO: carbon monoxide.
